# Tailoring IGZO Composition for Enhanced Fully Solution-Based Thin Film Transistors

**DOI:** 10.3390/nano9091273

**Published:** 2019-09-06

**Authors:** Marco Moreira, Emanuel Carlos, Carlos Dias, Jonas Deuermeier, Maria Pereira, Pedro Barquinha, Rita Branquinho, Rodrigo Martins, Elvira Fortunato

**Affiliations:** i3N/CENIMAT, Department of Materials Science, Faculty of Science and Technology, Universidade NOVA de Lisboa and CEMOP/UNINOVA, Campus de Caparica, 2829-516 Caparica, Portugal

**Keywords:** IGZO composition, solution combustion synthesis, transparent amorphous semiconductor oxides, low voltage operation

## Abstract

Solution-processed metal oxides have been investigated as an alternative to vacuum-based oxides to implement low-cost, high-performance electronic devices on flexible transparent substrates. However, their electrical properties need to be enhanced to apply at industrial scale. Amorphous indium-gallium-zinc oxide (a-IGZO) is the most-used transparent semiconductor metal oxide as an active channel layer in thin-film transistors (TFTs), due to its superior electrical properties. The present work evaluates the influence of composition, thickness and ageing on the electrical properties of solution a-IGZO TFTs, using solution combustion synthesis method, with urea as fuel. After optimizing the semiconductor properties, low-voltage TFTs were obtained by implementing a back-surface passivated 3-layer In:Ga:Zn 3:1:1 with a solution-processed high-к dielectric; AlO_x_. The devices show saturation mobility of 3.2 cm^2^ V^−1^ s^−1^, *I_On_*/*I_Off_* of 10^6^, *SS* of 73 mV dec^−1^ and *V_On_* of 0.18 V, thus demonstrating promising features for low-cost circuit applications.

## 1. Introduction

In recent years, the emergence of flexible electronics has increased scientific interest in transparent amorphous metal oxide thin-film transistors (TFTs) deposited at low temperatures on flexible substrates. These devices are expected to meet technological demands for a wide range of flexible electronic concepts, such as foldable displays or signal readout/processing circuitry integrated in smart surfaces [1,2]. The advent of printed electronics research has led to the development of solution processed oxides deposited by techniques such as spin coating [3] and ink-jet printing [4], as an economic alternative to vacuum-based techniques [2]. In this regard, solution-processed amorphous oxide semiconductors TFTs offer low-cost, high-throughput and large-area scalability [5,6]. However, with sol-gel methods, it is difficult to modulate oxygen conditions that are crucial to form oxygen vacancies, which are a source for free carriers; therefore, electrical properties are controlled by post-annealing or composition of the metal oxide [7]. Up until now, a variety of solution-produced transparent oxides such as zinc oxide (ZnO) [8], zinc-tin oxide (ZTO) [9] and indium-zinc oxide (IZO) [10] have been a matter of study. Nevertheless, indium-gallium-zinc oxide (IGZO) remains the most used oxide semiconductor and Table 1 summarizes the reported properties of solution based IGZO TFTs produced with different processing conditions.

The first reported IGZO solution TFTs were fabricated using high annealing temperatures (>400 °C) [11,12,13,15] in order to remove organic ligands groups from sol-gels, i.e., to convert completely metal-hydroxide (M–OH) species into metal-oxygen-metal (M–O–M). Nonetheless, high temperature annealing restricts the application of the films on most flexible polymeric substrates [21,22]. Some reports tested ~300 °C [17,18]; however, at lower temperatures, incomplete decomposition of the organic precursors might occur, and most of the M–OH species are not fully converted into M–O–M, severely affecting the semiconductor’s electrical performance [18,23]. In 2011, Marks et al. [24] reported, for the first time, a novel method to produce thin films at lower temperatures: the combustion synthesis method. By introducing an oxidizing agent (metal nitrates) and a fuel as reducing agent into a precursor solution, the potential of the oxide precursor is enhanced; when the film is annealed at 200–300 °C, a local highly exothermic chemical reaction initiates within the film, forming M–O–M lattice where the applied temperature acts only as reaction initiator [24,25]. Acetylacetone [19,26] and urea [27,28] are the most commonly used fuels in this method for different solution-based semiconductors. IGZO is applied mainly as semiconducting n-channel layer in TFTs, due to its high field-effect mobility, small subthreshold slope (SS), stability and good uniformity [29,30,31]. In^3+^ cations are the main element of conduction band and due to the overlap of their *5s* orbitals, IGZO exhibits high mobility, even in its amorphous form; Zn^2+^ contributes to stabilization and enhancement of electrical properties; and Ga^3+^ forms strong bonds with oxygen, controlling the carrier concentration so the material might act as a semiconductor, although this reduces the electron mobility compared to IZO [1,2,16]. Although there are a few reports regarding the effect of sol-gel IGZO composition on TFTs performance [14,15], the study on how electrical properties of solution combustion synthesis IGZO depend on material composition is still lacking. In this work, we discuss the influence of In:Ga:Zn cations ratios of combustion solution-processed IGZO, as well as the number of implemented layers on TFTs performance. Urea was chosen as fuel to use throughout this work, since it is more environment-friendly and less-expensive when compared to acetylacetone. Solution-based aluminum oxide (AlO_x_) dielectric was implemented in IGZO TFTs, as superior device performance can be achieved by combining a high-к oxide dielectric with a semiconductor material, namely, increased mobility and lower operation voltage compared to conventional SiO_2_ dielectric [32].

## 2. Experimental Section

### 2.1. Precursor Solution Development and Characterization

The metallic oxide precursor solutions were prepared by individually dissolving indium (III) nitrate hydrate (In(NO_3_)_3_·xH_2_O, Sigma, 99.9%, Darmstadt, Germany), gallium (III) nitrate hydrate (Ga(NO_3_)_3_·xH_2_O, Sigma, 99.9%, Darmstadt, Germany), zinc nitrate hexahydrate (Zn(NO_3_)_2_·6H_2_O, ACROS Organics, 98%, Geel, Belgium) and aluminum nitrate non-hydrate (Al(NO_3_)_3_.·9H_2_O, Carl Roth, ≥98%, Darmstadt, Germany) in 2-Methoxyethanol (2-ME) (C_3_H_8_O_2_, ACROS Organics, >99.5%, Geel, Belgium), to yield solutions with a concentration of 0.2 M. Urea (CO(NH_2_)_2_, Sigma, 98%, Darmstadt, Germany) was added as fuel to each precursor solution for the combustion reaction, with molar ratios between urea and indium nitrate, gallium nitrate, zinc nitrate and aluminum nitrate of 2.5:1, 2.5:1, 1.67:1 and 2.5:1, respectively, to guarantee the redox stoichiometry of the reaction (see Tables S1–S5 in the Supplementary Materials). All precursor solutions were magnetically stirred at 430 rpm for 1 h at room temperature in air environment. IGZO precursor solutions were prepared by mixing indium nitrate, gallium nitrate and zinc nitrate precursor solutions to yield In:Ga:Zn molar ratios of 1:1:1, 2:1:1, 2:1:2 and 3:1:1, all with a 0.2 M concentration. IGZO and AlO_x_ solutions were magnetically stirred at 430 rpm for at least 24 h at room temperature in air environment. All solutions were filtrated through 0.2 µm hydrophilic filters. Precursor solutions viscosity measurements were performed in a BROOKFIELD Cap 2000+ (Brookfield Engineering Laboratories, Inc., Middleboro, MA, USA) using a Cap01 spindle at 30 °C with a 500 rpm speed.

Thermal and chemical characterization of precursor solutions were performed by differential scanning calorimetry (DSC) and thermogravimetry (TG) and Fourier transform-infrared spectroscopy (FTIR). DSC and TG analysis of dried precursor solutions were performed under air atmosphere up to 500 °C with a 10 °C min^−1^ heating rate in an aluminum crucible with a punctured lid using a simultaneous thermal analyzer, Netzsch (TG-DSC—STA 449 F3 Jupiter, Selb, Germany). FTIR spectroscopy characterization of IGZO solutions was performed using a Thermo Nicolet 6700 Spectrometer (Waltham, MA, USA) equipped with a single bounce diamond crystal Attenuated Total Reflectance (ATR) sampling accessory (Smart iTR). The spectra were acquired with a 4 cm^−1^ resolution in the range of 4000–525 cm^−1^ with a 45° incident angle.

### 2.2. IGZO Film Deposition and Material Characterization

Prior to deposition all substrates (p^+^Si with a 100 nm thermally grown SiO_2_ layer, Si wafer and soda-lime glass, 2.5 × 2.5 cm^2^) were cleaned in an ultrasonic bath at 60 °C in acetone for 15 min, then in isopropyl alcohol (IPA) for 15 min. Subsequently, the substrates were cleaned with deionized water (DIW) and dried under N_2_, followed by a 15 min ultraviolet (UV)/ozone surface activation step using a PSD-UV Novascan system (Ames, IA, USA). IGZO thin films were deposited onto SiO_2_ substrates by sequentially spin coating one to three layers of IGZO precursor solution for 35 s at 3000 rpm (Laurell Technologies, North Wales, PA, USA), followed by an immediate hot plate annealing at 300 °C for 30 min in air after each layer to ensure the exothermic reaction. The AlO_x_ dielectric precursor solution was spin coated at 2000 rpm for 35 s onto Si substrates and annealed at 300 °C for 30 min. FTIR spectroscopy characterization of thin films deposited on Si substrates was performed the same way as used for IGZO precursor solutions. The structure of the films was assessed by grazing angle X-Ray diffraction (GAXRD), using a X’Pert PRO PANalytical (Royston, UK) diffractometer with Cu K*α* line radiation (λ = 1.540598 Å) and an incidence angle of the X-ray beam fixed at 0.75°, in the range of 20° to 50° (2θ). Surface morphology of the thin films was studied by scanning electron microscopy (SEM, Zeiss Auriga Crossbeam electron microscope) ( Oberkochen, Germany) and atomic force microscopy (AFM, Asylum MFP3D, Asylum Research, Santa Barbara, CA, USA). Electron dispersive X-ray spectroscopy (EDS) was performed to study the chemical composition of the thin films. Optical characterization of the thin films was obtained with a Perkin Elmer lambda 950 UV/visible (Vis)/near infrared (NIR) (Llantrisant, UK) spectrophotometer, by measuring transmittance variation in a wavelength range from 200 to 2500 nm. Spectroscopic ellipsometry was used to measure thickness and band gap energy of thin films deposited on Si substrates, with an energy range from 1.5 to 5.5 eV and an incident angle of 45° using a Jobin Yvon Uvisel system (Chilly-Mazzarin, France). DELTAPSI software (v2.6.6.212, Horiba, Bensheim, Germany) was used to modulate the acquired data, and the fitting procedure was done pursuing the minimization of the error function (χ^2^). X-ray photoelectron spectroscopy (XPS) of IGZO thin films was measured with a Kratos Axis Supra (Manchester, UK), using monochromated Al Kα irradiation (1486.6 eV). The detail spectra of the surfaces were acquired with an X-ray power of 225 W and a pass energy of 10 eV. Depth profiles were done using argon clusters of 500 atoms and 10 keV, scanned over an area of 1.5 × 1.5 mm^2^ and a time per etch step of 100 s. The cluster mode was used in order to limit damage to the film introduced by the argon beam with respect to a conventional monoatomic mode. Here, the XPS acquisition parameters were 300 W and 40 eV pass energy, and an aperture was used to limit the measurement spot to 110 μm in diameter.

### 2.3. TFTs/Devices Fabrication and Characterisation

TFTs were produced in a staggered bottom-gate, top-contact structure by spin coating IGZO thin films onto 100-nm-thick thermal SiO_2_ (*C_i_* = 35 nF·cm^−2^) or onto spin-coated 20-nm-thick AlO_x_ (*C_i_* = 306 ± 2 nF·cm^−2^), both on Si wafers. Aluminum (Al) source and drain electrodes (80 nm thick) were deposited on IGZO films via a shadow mask by thermal evaporation, with channel width (W) and length (L) of 1400 µm and 100 µm, respectively (W/L = 14). A post-annealing step was performed on a hot plate for 1 h at 120 °C in air environment. Optimized IGZO/AlO_x_ devices were patterned by standard photolithographic processes (W/L = 160/20) and passivated with 1 µm thick parylene. Electrical characterization was performed by measuring current-voltage characteristics of the devices using a semiconductor parameter analyzer (Agilent 4155C, Agilent Technologies, Santa Clara, CA, USA) attached to a microprobe station (Cascade M150, FormFactor, Livermore, CA, USA), inside a Faraday cage, in the dark and at room temperature.

Transfer curves were performed in double sweep mode and used to extract turn-on voltage (*V_On_*), threshold voltage (*V_T_*), hysteresis (*V_Hyst_*), subthreshold slope (*SS*), mobility in saturation regime (*μ_Sat_*) and on/off current ratio (*I_On_*/*I_Off_*). A gate-to-source voltage (*V_GS_*) from −10 to 20 V and a drain-to-source voltage (*V_DS_*) of 20 V were applied. SS was estimated by [33]:

$$SS={\left({\frac{d\log\left(I_{DS}\right)}{dV_{GS}}|}_{\max}\right)}^{-1}$$

*V_T_* was derived from a linear fitting $\sqrt{I_{DS}}\;vs.\;V_{GS}$ in the saturation region [33]:

$$I_{DS}=\frac{WC_{i}}{2L}\mu_{Sat}{\left(V_{GS}-V_{T}\right)}^{2}$$

where *W* and *L* are the channel width and length, and *C_i_* is the gate dielectric capacitance per unit area. *μ_Sat_* was obtained by [33]:

$$\mu_{Sat}={\left(\frac{\partial \sqrt{I_{DS}}}{\partial V_{GS}}\right)}^{2}\frac{2L}{WC_{i}}$$

Positive gate bias stress tests were performed on IGZO/AlO_x_ TFTs using a semiconductor parameter analyzer (Keysight 4200SCS, Penang, Malaysia) and probe station (Janis ST-500, Woburn, MA, USA) under air environment by applying a constant gate voltage (0.5 MV·cm^−1^ electric field) for one hour.

## 3. Results and Discussion

### 3.1. Precursor Solutions and Thin Films Characterization

Solution combustion synthesis is an efficient method to obtain high-quality thin films at lower temperatures than sol-gel, by initiating an exothermic reaction between an oxidizer (usually nitrates) and an organic fuel acting as reducing agent. The generated localized energy efficiently converts the metal nitrates precursors into oxides [24,34].

**Figure 1:**
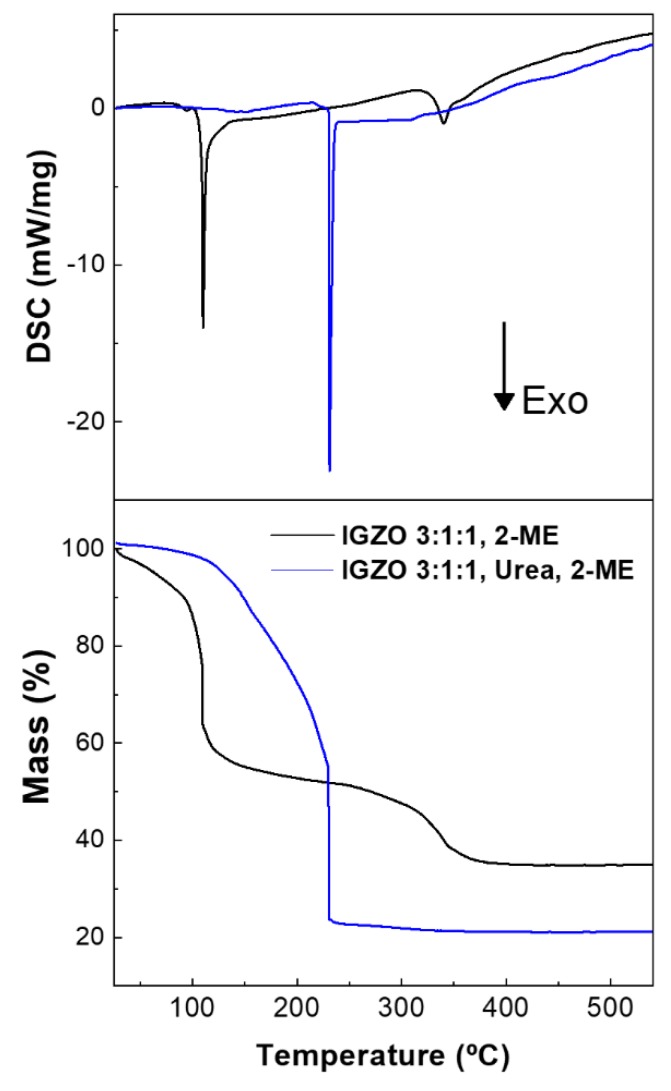
Differential scanning calorimetry (DSC)-thermogravimetry (TG) analysis of the IGZO (3:1:1) precursor solution with 2-ME as solvent and using with or without urea as fuel.

The thermal characterization of the precursor solutions is relevant to evaluate the decomposition of metal oxides and the combustion reaction ignition temperature. Figure 1 illustrates DSC-TG data of IGZO 3:1:1 0.2 M solutions with and without urea.

**Figure 2:**
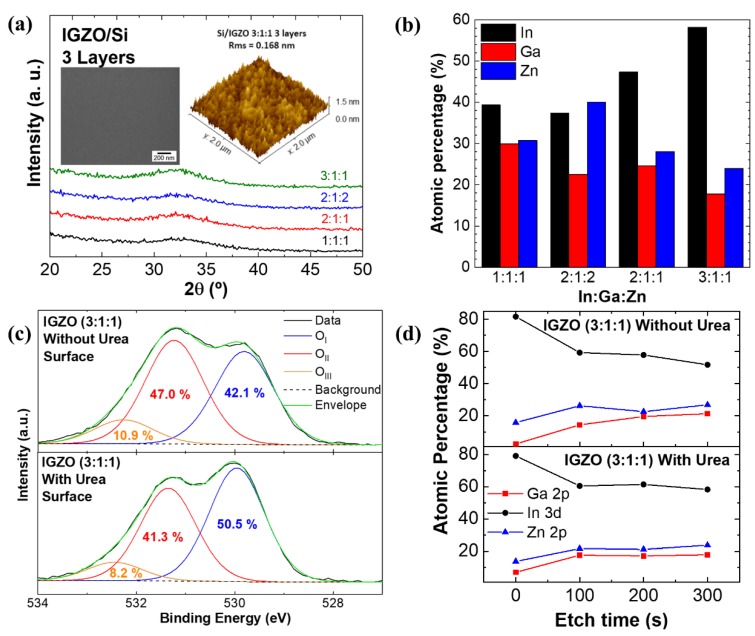
(**a**) X-Ray diffraction (XRD) of combustion IGZO films with different In:Ga:Zn ratio; inset shows the scanning electron microscopy (SEM) surface image and atomic force microscopy (AFM) topography of 3-layer IGZO 3:1:1; (**b**) atomic concentration (%) of each metallic cation in 3-layer combustion IGZO films with different In:Ga:Zn ratio determined by electron dispersive X-ray spectroscopy (EDS) analysis; (**c**) X-ray photoelectron spectroscopy (XPS) surface spectra of IGZO 3:1:1 thin films produced with and without urea (**d**) and In:Ga:Zn atomic percentage after argon cluster etching (0–300 s).

The intense exothermic peaks, accompanied by a significant weight loss, correspond to the combustion reaction of residual fuel in the formation of IGZO thin films. For the precursor solution without urea, two exothermic peaks are observed at 110 °C and 340 °C, which can indicate the formation of two distinct materials due to a non-uniform distribution of the metal cations within the gel phase of the reaction. This has also been observed for the formation of other multicomponent oxides, such as ZTO, where the presence of more than one metal cation can lead to multistep synthesis and consequently ununiform material [35]. Thus, the complete conversion of the precursor without urea requires temperatures above 300 °C. When urea is used as fuel, only one exothermic peak is observed at 230 °C, which indicates that the IGZO formation occurs in one step, thus contributing to the uniform cation distribution. This conclusion is corroborated by comparison of In:Ga:Zn ratios by EDS and XPS analysis, as discussed further below (Figure 2b–d).

In the solution combustion precursor, no weight loss was observed above 230 °C, suggesting that this annealing temperature is enough to eliminate all organics in IGZO films. Nevertheless, for IGZO films deposition the annealing was perform at 300 °C in order to assure the complete formation of M–O–M. To study the influence of composition and thickness in produced IGZO films’ properties, combustion precursor solutions were prepared with In:Ga:Zn ratio of 1:1:1; 2:1:1; 2:1:2 and 3:1:1 and 1-, 2- and 3-layered films IGZO thin films were deposited by spin-coating.

FTIR spectra of all IGZO films were performed after annealing at 300 °C and compared to spectra of precursor solutions which confirm the removal of organic compounds and the presence of M-O bonds in thin films after annealing (see Figure S1 and Table S6 in the Supplementary Materials).

IGZO films thickness was assessed by spectroscopic ellipsometry for all conditions. As expected, film thickness does not vary significantly for different composition as for all precursor solutions the viscosity is 2.30 ± 0.04 cP (Table S7), since concentration was maintained constant at 0.2 M. Additionally, film thickness increases almost linearly with the number of deposited layers with d ≈ 14 nm for 1-layer films; d ≈ 27 nm for 2-layer films and d ≈ 37 nm for 3-layer films (Table S8). Optical characterization shows typical average transmittance in the visible range of ~88% (Figure S2) as expected for IGZO thin films.

Optical bandgap energy (E_g_) calculation by spectroscopic ellipsometry for combustion IGZO films with different In:Ga:Zn ratio (Table S9) reveals higher E_g_ for IGZO 1:1:1 (Ga-rich, E_g_ = 3.68 ± 0.03 eV), which is expected due to the higher E_g_ of GaO_x_ compared to InO_x_ and ZnO, whereas for the remaining compositions E_g_ = 3.45 ± 0.06 eV which is in agreement with reported values of IGZO films [29].

XRD, AFM and SEM analysis were performed to assess structural and morphological characteristics of the thin films. XRD analysis of solution processed IGZO was obtained by spin coating three layers on Si substrates. The absence of diffraction peaks indicates that no long-range order is present, as expected for multicomponent oxides, and amorphous films are obtained up to 300 °C (Figure 2a).

SEM surface images and AFM deflection (Figure 2a inset) show that smooth and uniform films are obtained regardless of processing conditions. The films roughness was determined from the AFM height profile of a 2 × 2 μm^2^ area scan with rms roughness being lower than 0.3 nm for all films (Figure S3), as required for the integration in electronic devices.

Atomic percentage of metal cations was determined for combustion IGZO films by EDS analysis to determine films stoichiometry for different In:Ga:Zn ratio. Figure 2b shows that in general the films stoichiometry matches the In:Ga:Zn ratio of IGZO precursor solutions with a slight Ga deficiency for 2:1:1 and 3:1:1 films.

X-ray photoelectron spectroscopy (XPS) was performed to evaluate the structure of IGZO films produced with and without urea. Figure 2c shows high resolution spectra of the initial films’ surfaces. Differential charging occurred during this measurement, thus, the spectra were charge referenced to C 1 s at 284.8 eV a posteriori. The O 1s spectra of the films’ surfaces are deconvoluted into three main peaks. The first component (O_I_) at its lowest binding energy, 529.9 ± 0.1 eV, corresponds to M–O–M bonds [36]. The second component (O_II_), centered at 531.3 ± 0.1 eV, is either associated with M–O–M bonds at the surface or undercoordinated oxygen [37]. The third component (O_III_), centered at 532.3 ± 0.1 eV, is related to water and organic species adsorbed on the surface [36]. The two films have an identical C 1 s emission (not shown here), confirming a similar amount of contamination by adventitious carbon. Hence, it can be concluded that the addition of urea during the synthesis promotes the formation of M–O–M bonds at the surface. In order to address the volume of the films, XPS depth profiles were performed, given in Figure 2d. An argon cluster mode was chosen in order to induce less damage to the material than with a conventional monoatomic mode. The motivation to do depth profiles came from the cation stoichiometries observed at the surface (given in Figure 2d after 0 s of etching), which show that both films’ surfaces are highly deficient in gallium, particularly the film produced without urea. For both films prepared with and without urea, the cation stoichiometries tend to match the EDS results of Figure 2b scanning the films’ thicknesses towards the substrate interface. However, the cation stoichiometry of the film prepared with urea matches exactly the EDS results already after the first etching step and is maintained throughout the films’ thickness. On the contrary, the gallium content of the film prepared without urea continuously increased with further etching. Two conclusions can be made from these results: first, the as-deposited surfaces of the IGZO films (with and without urea) are generally poor in gallium; second and most importantly, combustion synthesis using urea as fuel promotes film formation with a uniform cation distribution throughout the thickness, which is in line with the single exothermic peak in the DSC analysis in Figure 1.

Note that after argon cluster etching, only the first two O 1s components are observed and the third peak originating from adsorbates is no longer present (see Supporting Information Figure S4). This supports the assignment of the O 1s components made above, with the O_II_ component partially and the O_III_ component entirely related to adsorbed surface species.

### 3.2. Electrical Characterization of IGZO Thin Film Transistors (TFTs)

**Figure 3:**
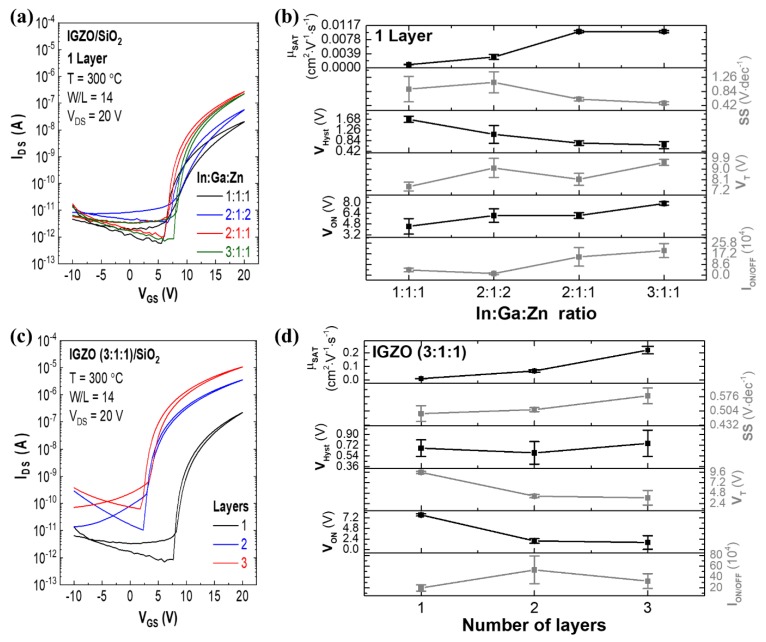
Transfer characteristics of IGZO thin-film transistors (TFTs) (**a**) with a different In:Ga:Zn ratio and (**b**) respective electrical parameters; (**c**) with 1-, 2- and 3-layer IGZO 3:1:1 TFTs and (**d**) their respective electrical parameters.

Electrical characterization of solution processed IGZO/SiO_2_ TFTs was performed by measuring the transfer characteristics of the devices in ambient conditions in the dark to study the influence of semiconductor composition ratio and number of layers in device performance (Figure 3).

Figure 3a shows transfer characteristics of IGZO TFTs with different compositions, and IGZO TFTs with variation of layers; the statistics of the extracted parameters are represented in Figure 3b. For In-rich composition IGZO 3:1:1, the on/off current ratio (*I_On_*/*I_Off_*) and saturation mobility (*μ_Sat_*) increases one order of magnitude compared to IGZO 2:1:1 and 1:1:1. Indium cations constitute the main element of conduction band minimum (CBM) in these amorphous structures, where potential barriers arising from random distribution of zinc and gallium cations exist. Thus, by increasing indium content, the potential barriers derived from structural randomness decrease, enhancing carrier transport. In IGZO 1:1:1 the values of *I_On_*/*I_Off_* and *μ_Sat_* are lower as the higher gallium content helps suppress free carrier generation by forming stronger chemical bonds with oxygen when compared to zinc and indium cations. Therefore, gallium and zinc content must be tailored to guarantee amorphous films [16,38]. Still, it is relevant to notice from Figure 3a that off-current is not being significantly affected by the different IGZO compositions, being governed by the gate-to-source leakage current (*I_GS_*), as expected for a TFT. However, this can also be related with the very low thickness of IGZO with 1 layer (d ≈ 14 nm), allowing for the depletion region arising from the atmosphere interaction with the IGZO back-surface to be extended through the entire IGZO films thickness [39]. The results obtained for different IGZO thickness, discussed below, shed light into this phenomenon.

To understand the thickness influence, a different number of layers (1, 2 and 3) were studied in IGZO 3:1:1 TFTs (Figure 3c,d). It is evident the negative shift on *V_On_* and *V_T_* and a higher *I_Off_* with the increasing number of spin coated layers, due to higher free carrier concentration (*N*) in the bulk of the thicker active layer, leading to charge accumulation in the semiconductor/dielectric interface; therefore the conductive channel forms at lower *V_GS_* values [39]. The better electrical performance of 3-layer films can also be associated with lower porosity as with each layer deposited defects caused by gaseous products release are decreased and film densification is enhanced.

Optimized electrical performance was obtained for 3-layer IGZO 3:1:1 TFTs. As such, and to further assess the effect of urea in device performance, 3-layer TFTs were also produced using IGZO 3:1:1 precursor solution without urea (Figure S5 in Supporting information). The later devices show overall poor electrical performance with high hysteresis and low stability, thus confirming the crucial role of urea in the proper formation of IGZO at temperatures ≤300 °C.

**Figure 4:**
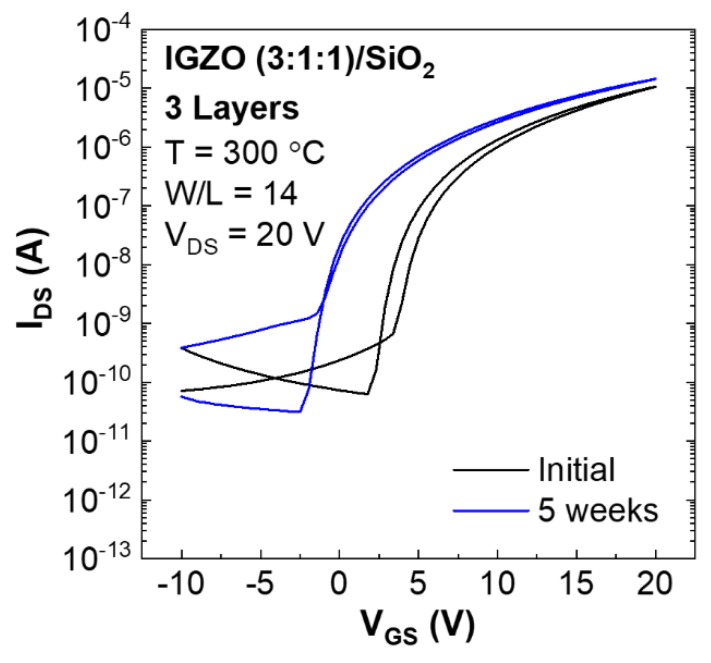
Transfer characteristics of a 3-layer IGZO 3:1:1 TFT as deposited and after 5 weeks.

Figure 4 shows transfer characteristics of 3-layer IGZO (3:1:1) TFTs as deposited and after 5 weeks to assess device ageing. Overall device performance (*I_On_*/*I_Off_*, SS and *μ_Sat_*) was maintained however *V_On_* and *V_T_* show a slight negative shift over time associated to the increase of carrier density which results in the rise of oxygen vacancy concentration in the channel [40].

Fully solution based TFTs were produced by combining optimized 3-layer IGZO 3:1:1 with high-к solution process dielectric AlO_x_ to enable low voltage operation. Capacitance variation with frequency of Si/AlO_x_/Al MIS devices is depicted in Figure S6, where *C_i_* = 306 ± 2 nF was determined for AlO_x_ at 100 Hz. IGZO/AlO_x_ TFTs were patterned (W/L = 160/20) and passivated with chemical vapor deposited parylene.

**Figure 5:**
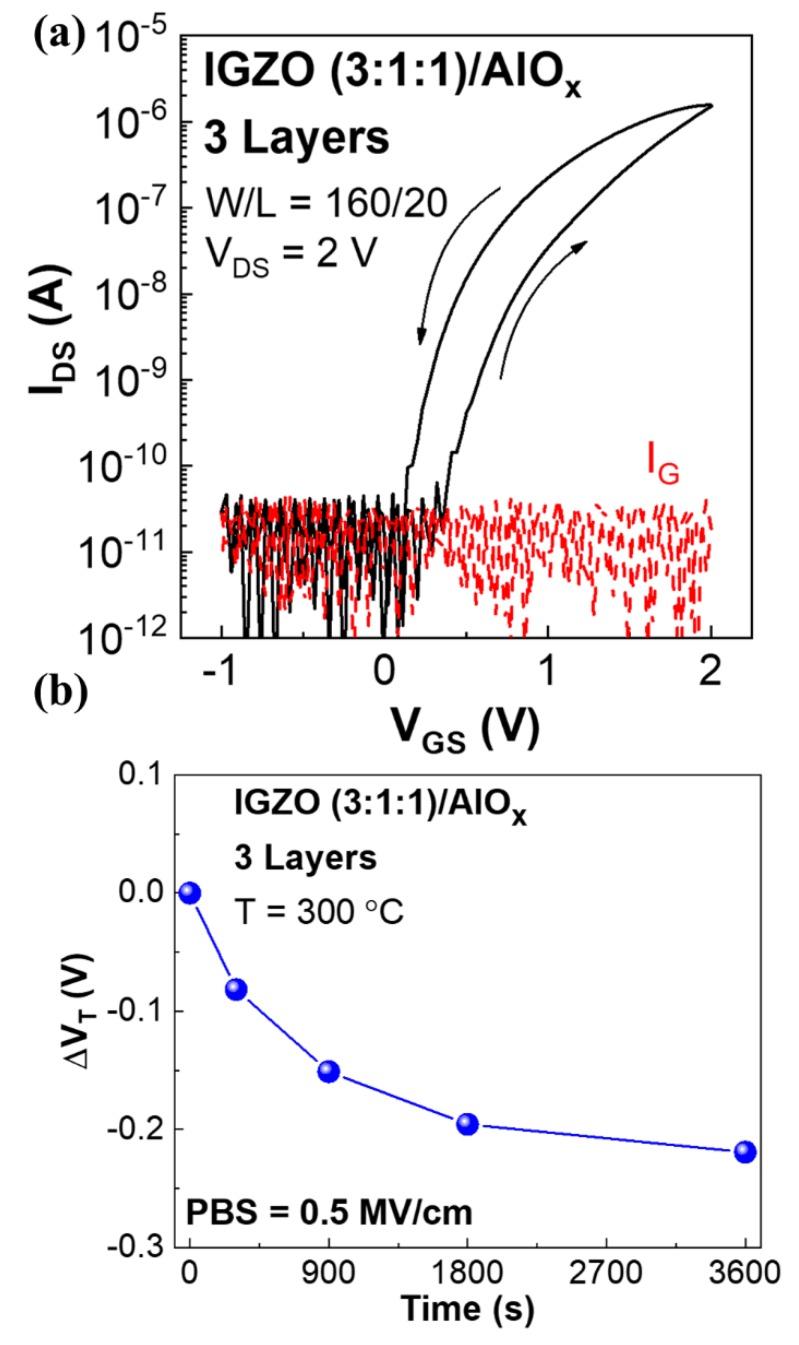
(**a**) Transfer characteristics of a fully solution-based passivated 3-layer IGZO 3:1:1/AlO_x_ TFT; (**b**) threshold voltage variation (ΔV_T_) under positive gate bias stress (PBS) (0.5 MV·cm^−1^) for 1 h in air environment.

Electrical characterization of fully solution-based 3-layer IGZO 3:1:1/AlO_x_ TFTs is depicted in Figure 5. TFTs transfer characteristics (Figure 5a) were obtained by varying *V_GS_* from −1 to 2 V for *V_DS_* of 2 V. The fully solution-based devices demonstrate enhanced performance when compared to SiO_2_ non-passivated TFTs namely, *I_On_*/*I_Off_* = 10^6^, *μ_Sat_* = 3.2 cm^2^ V^−1^ s^−1^, *SS* = 73 mV dec^−1^, *V_On_* = 0.18, *V_T_* = 0.63 V and *I_GS_*.

The devices operational stability under positive gate bias stress (PBS) was studied in air environment by applying a constant gate voltage equivalent to electrical field of 0.5 MV·cm^−1^, while keeping the source and drain electrodes grounded. The transfer characteristics of 3-layer IGZO (3:1:1)/AlO_x_ TFTs were obtained in saturation regime (*V_DS_* = 2 V) at selected times during stress (Figure S7 in the Supplementary Materials) and the threshold voltage variation (Δ*V_T_*) with time during PBS is shown in Figure 5b. The devices show a negative threshold voltage shifts under PBS, which was previously reported for IGZO TFTs using solution processed and sputtered high-κ dielectrics by our group [34,41]. The abnormal shift in *V_T_* when applying PBS is associated to the hydrogen release from residual AlO-H bonds in the AlO_x_ gate dielectric and their migration to the IGZO channel. By diffusing the hydrogen atoms in the channel, a negative Δ*V_T_* is induced through electron doping power-law time dependence [42]. Initially *V_T_* shifts abruptly however after 30 min the devices stabilize with maximum Δ*V_T_* = −0.22 V after 1 h of PBS.

## 4. Conclusions

In summary, we clearly demonstrated the importance of IGZO composition and number of layers in combustion solution based IGZO TFTs. The use of urea as fuel is crucial to produce high quality IGZO films at lower temperature and assure that the precursors In:Ga:Zn ratio is maintained in the films. Indium content plays a major role to achieve enhanced electrical properties and 3-layer films show improved densification. Fully solution processed TFTs with low operation voltage were achieved with optimized 3-layer IGZO (3:1:1) active channel layer and AlO_x_ high-к dielectric. These devices demonstrate enhanced dielectric-semiconductor interface (*SS* = 73 mV·dec^−1^) and saturation mobility of 3.2 cm^2^ V^−1^ s^−1^ with good stability over time. These results have been proved to be reproducible encouraging the use of fully solution based IGZO TFTs for low-cost electronic applications.

## Figures and Tables

**Table 1:** Solution-based indium gallium zinc oxide (IGZO) thin film transistors produced at T ≥ 300 °C by spin-coating deposition of 2-methoxyethanol (2-ME) based precursors.

Year	Fuel	T_max_ (°C)	W/L	Dielectric (Technique)	In:Ga:Zn Ratio	*µ_SAT_* (cm^2^/Vs)	*SS* (V/dec)	*I_On_*/*I_Off_*	*V_on_* (V)	*V_GS_* (V)
2008 [11]	No	450	1000/150	SiN_x_ (PECVD)	1:1:2	0.96	1.39	10^6^	~5	−15 to 55
2009 [12]		400	1000/150	SiN_x_ (PVD)	1:1:2	0.56 (μ_ef_)	2.81	4.6 × 10^6^	5	−30 to 30
					3:1:2	0.90 (μ_ef_)	1.16	3.8 × 10^6^	~0	
					5:1:2	1.25 (μ_ef_)	1.05	4.1 × 10^6^	−10	
2009 [13]		400	100/50	SiO_2_	2:1:2	2 (μ_ef_)	-	10^5^	-	−40 to 40
2010 [14]		400	1000/90	ATO (ALD)	3:1:1	5.8 (μ_lin_)	0.28	6 × 10^7^	~0	−10 to 30
2010 [15]		500	200/20	SiO_2_ (Thermal oxidation)	4:1:5	1.13	2.5	-	-	−30 to 40
2010 [7]		450	1000/150	SiN_x_	3:1:2	0.86 (μ_lin_)	0.63	10^6^	~0	−30 to 30
2010 [16]		300	1000/100	SiO_2_	63:10:27	0.2	-	10^5^	~−15	−40 to 40
2011 [17]		300	500/100	SiO_2_	5:1:2	0.003	2.39	4.5 × 10^4^	-	−20 to 30
2013 [18]		300	1000/100	SiO_2_ (Thermal oxidation)	62:5:23	1.73 (μ_ef_)	0.32	10^7^	11	−10 to 40
2013 [19]	Yes (acac)	300	5000/100	SiO_2_ (Thermal Oxidation)	80:10:10	5.43	-	10^8^	-	0 to 100
2019 [20]		300	1000/100	SiO_2_	10:1:3	1.62	0.03	10^6^	~0	−40 to 80
2019 [3]	No	350	n.d./100	SiO_2_	68:10:22	0.72	0.68	10^6^	~0	−30 to 30
This work	Yes (urea)	300	160/20	AlO_x_	3:1:1	3.2	0.073	10^6^	0.18	−1 to 2

W/L: Width/Length; PECVD: plasma enhanced vapor deposition PVD: physical vapor deposition; ATO: aluminum-doped tin oxide; ALD: atomic layer deposition; acac: acetylacetone; n.d.: not defined.

## Supplementary Materials

The following are available online at https://www.mdpi.com/2079-4991/9/9/1273/s1, Table S1: Redox reactions regarding this work, Table S2: Overall oxide formation reaction considering metal nitrate reduction and urea oxidation reactions, Table S3: Calculation of oxidizing and reducing valence of reagents by the Jain method, Table S4: umber of moles of urea per mole of oxidant to ensure stoichiometry (φ = 1) of the redox reaction, Table S5: Stoichiometric overall oxide formation reactions, Figure S1: (a) FTIR spectra of IGZO solutions; (b) 3-layer IGZO thin films on Si substrates, after annealing at 300 °C for 30 min, Table S6: Characteristic absorbance peaks and associated vibrational modes of the corresponding chemical bonds for analyzed FT-IR spectra of IGZO solutions, Table S7: Viscosity of combustion IGZO precursor solutions with different In:Ga:Zn ratio, Table S8: Combustion IGZO films’ thickness measured by spectroscopic ellipsometry, Table S9: Bandgap energy (Eg) of combustion IGZO thin films determined by spectroscopic ellipsometry, Figure S2: Transmittance measurements of combustion of 1-, 2- and 3-layer IGZO 3:1:1, Figure S3: AFM deflection images (2 × 2 µm2) of 1-, 2- and 3-layer IGZO thin films with different In:Ga:Zn ratio (a) 1:1:1, (b) 2:1:2, (c) 2:1:1 and d) 3:1:1, Figure S4: Deconvoluted O 1s spectra of the XPS depth profile after 0 s, 100 s and 200 s argon cluster etching, Figure S5: Transfer characteristics of 3-layer 3:1:1 IGZO TFTs produced using IGZO precursor solutions with and without urea as fuel, Figure S6: Capacitance-frequency measurements of Si/solution-based AlOx/Al MIS device, Figure S7: Transfer characteristics of 3-layer IGZO (3:1:1)/AlOx TFT when a positive gate bias stress (PBS) of 0.5 MV∙cm^−1^ is applied over time.
