# Supplementary material for: Tailoring IGZO Composition for Enhanced Fully Solution-Based Thin Film Transistors
Source: Nanomaterials (Basel). 2019 Sep 6;9(9):1273. doi: 10.3390/nano9091273 (PMC6781023; doi:10.3390/nano9091273)
Supplement: Supplementary file 1 [file nanomaterials-09-01273-s001.pdf]

Tailoring IGZO Composition for Enhanced Fully Solution-Based Thin Film Transistors

Marco Moreira, Emanuel Carlos, Carlos Dias, Jonas Deuermeier, Maria Pereira, Pedro Barquinha *, Rita Branquinho *, Rodrigo Martins and Elvira Fortunato

i3N/CENIMAT, Department of Materials Science, Faculty of Science and Technology, Universidade NOVA de Lisboa and CEMOP/UNINOVA, Campus de Caparica, 2829-516 Caparica, Portugal
* Correspondence: pmcb@fct.unl.pt (P.B.); ritasba@fct.unl.pt (R.B.)

The supporting information contains relevant data related to the characterisation of IGZO thin films and devices developed in this work. Table S1–S5 show redox reactions of oxide formation, FTIR spectra of IGZO precursor solutions and thin films are depicted in Figure S1 and Table S6. Viscosity of combustion precursor solutions are shown in Table S7. IGZO films thickness (Table S8), bandgap energy (Table S9) and optical transmittance (Figure S2) are also presented. AFM deflection images of IGZO thin films are depicted in Figure S3. Figure S4 depictes XPS O1s spectra after argon cluster etching for 0, 100 and 200 s on IGZO surface. Figure S5 compares transfer characterisitics of IGZO TFTs produced with and without urea. Figure S6 shows the capacity-frequency of the solution processed AlO_x dielectric used in fully solution based IGZO TFTs. Figure S7 compiles transfer characteristics of IGZO TFTs under positive gate bias stress (PBS).

Redox Reactions

Solution combustion synthesis involves the reduction of metallic nitrates reactions, which are converted into metal oxides, and the oxidation reactions of fuel by the nitrate ions (Table S1). Note that the metallic salts precursor solutions are balanced with urea used as fuel for the reaction.

Table S1. Redox reactions regarding this work.

Reduction reactions	
Indium nitrate hydrate	$2In(NO_3)_3 \cdot H_2O \rightarrow In_2O_3 + 2H_2O + 3N_2 + \left(\frac{15}{2}\right) O_2$
Gallium nitrate hydrate	$2Ga(NO_3)_3 \cdot H_2O \rightarrow Ga_2O_3 + 2H_2O + 3N_2 + \left(\frac{15}{2}\right) O_2$
Zinc nitrate hexahydrate	$Zn(NO_3)_2 \cdot 6H_2O \rightarrow ZnO + 6H_2O + N_2 + \left(\frac{5}{2}\right) O_2$
**Oxidation Reactions**	
Urea	$CO(NH_2)_2 + \left(\frac{3}{2}\right) O_2 \rightarrow 2H_2O + CO_2 + N_2$
2-Methoxyethanol	$C_3H_8O_2 + 4O_2 \rightarrow 4H_2O + 3CO_2$

The overall combustion reaction involves the combination of reduction and oxidation reaction; gaseous products are formed and released, including H_2O, N_2, CO_2 and O_2 [1]. These reactions are represented in Table S2.

Table S2. Overall oxide formation reaction considering metal nitrate reduction and urea oxidation reactions.

Oxide	Overall reaction
In_2O_3	$2In(NO_3)_3 \cdot H_2O + CO(NH_2)_2 \rightarrow In_2O_3 + 4H_2O + CO_2 + 4N_2 + 6O_2$

Ga_2O_3	$2Ga(NO_3)_3 \cdot H_2O + CO(NH_2)_2 \rightarrow Ga_2O_3 + 4H_2O + CO_2 + 4N_2 + 6O_2$
ZnO	$Zn(NO_3)_2 \cdot 6H_2O + CO(NH_2)_2 \rightarrow ZnO + 8H_2O + CO_2 + 2N_2 + O_2$

By using the Jain method, the stoichiometric proportion of oxidiser and fuel can be calculated in order to obtain the molar ratio of reactants and ensure the redox stoichiometry of the reaction, given by Equation 1 [2].

$$\varphi = \frac{RV}{OV} n \quad \text{(Equation 1)}$$

Where φ is the fuel/oxidiser ratio, RV and RO are reducing valence and oxidising valence respectively, and *n* is the number of moles of fuel per mole of oxidant. To have a ideal stoichiometric composition (φ =1) of redox mixture is obtained, because noadditional oxygen is required to complete the reaction. Indium and gallium and aluminium, zinc, carbon and hydrogen count all as reducing agents with corresponding valences of +3, +2, +4 and +1, respectively. Oxygen and nitrogen are considered oxidiser agents with valence of –2 and 0, respectively. Calculations of oxidising and reducing valences values are represented in Table S3. Note that for metal nitrates hydrates, water molecules do not affect the overall valence [1].

Table S3. Calculation of oxidizing and reducing valence of reagents by the Jain method.

	Reagent	Calculation	Total
**Oxidizing valence (OV)**	$In(NO_3)_3$	$3 + (3 \times 0) + [3 \times 3 \times (-2)]$	-15
	$Ga(NO_3)_3$	$3 + (3 \times 0) + [3 \times 3 \times (-2)]$	-15
	$Zn(NO_3)_2$	$2 + (2 \times 0) + [2 \times 3 \times (-2)]$	-10
**Reducing valence (RV)**	$CO(NH_2)_2$	$4 + (-2) + (2 \times 0) + (2 \times 2 \times 1)$	$+6$

Therefore, the number of moles needed to ensure stoichiometry of the redox reaction can be determined, with the values being shown in Table S4.

Table S4. Number of moles of urea per mole of oxidant to ensure stoichiometry (φ = 1) of the redox reaction.

Precursor	n
Indium nitrate hydrate	5/2
Gallium nitrate hydrate	5/2
Zinc nitrate hexahydrate	5/3
Aluminium nitrate non-hydrate	5/2

Finally, the global reactions are represented in Table S5 considering the number of moles to ensure the stoichiometry of the redox reaction.

Table S5. Stoichiometric overall oxide formation reactions.

Oxide	Overall reaction
In_2O_3	$2In(NO_3)_3 \cdot 2H_2O + 5CO(NH_2)_2 \rightarrow In_2O_3 + 14H_2O + 5CO_2 + 8N_2$
Ga_2O_3	$2Ga(NO_3)_3 \cdot 2H_2O + 5CO(NH_2)_2 \rightarrow Ga_2O_3 + 14H_2O + 5CO_2 + 8N_2$

ZnO	$3Zn(NO_3)_2 \cdot 6H_2O + 5CO(NH_2)_2 \rightarrow 3ZnO + 28H_2O + 5CO_2 + 8N_2$

FT-IR analysis of precursor solutions and IGZO thin films

Figure S1 depicts the FT-IR spectra of IGZO solutions and thin films for each molar ratio studied. Most of the vibration bands in precursor solutions (Figure. S1a) are expected for 2-methoxyethanol, as identified in Table S6 [3,4].

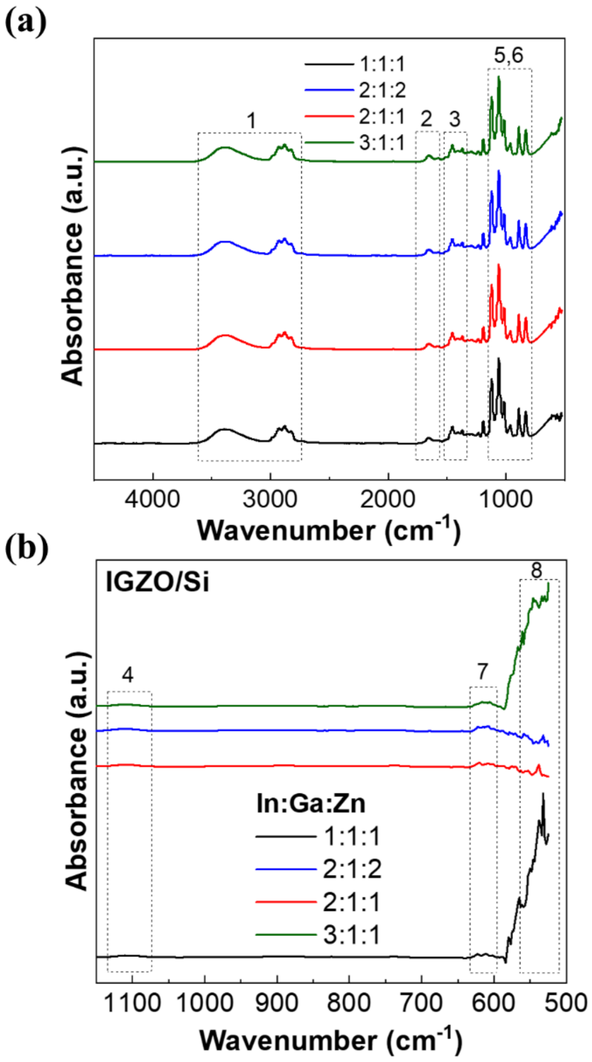

Figure S1. (**a**) FTIR spectra of IGZO solutions; (**b**) 3-layer IGZO thin films on Si substrates, after annealing at 300 °C for 30 min.

Table S6. Characteristic absorbance peaks and associated vibrational modes of the corresponding chemical bonds for analyzed FT-IR spectra of IGZO solutions.

Number	Position (cm^{-1})	Vibration Mode	Chemical Bond
**1**	3500	Stretching	M-OH
**2; 5**	1620 and 1015	Bending	M-OH
**3**	1388	Stretching	NO_3^-
**4**	1107	Stretching	Si-O
**6**	833	Bending	NO_3^-

7; 8	620, 509	Stretching	M-O

FTIR spectra of 3-layer IGZO thin films spin coated on Si substrates after being annealed at 300 °C for 30 min were obtained, with correction of the atmospheric contribution performed for all spectra (Figure. S2b). Si-O peak is observed at 1107 cm^{-1}, which is related with Si substrate; two M-O stretching vibration peaks at 620 cm^{-1} and 509 cm^{-1}, are observed (although only the increase of the latter peak is observed due to the range measurement of the equipment), confirming the presence of M-O bonds in thin films after annealing.

Table S7. Viscosity of combustion IGZO precursor solutions with different In:Ga:Zn ratio.

Solution	Viscosity (cP)
2-metoxyethanol	1.45±0.07
IGZO 1:1:1	2.34±0.08
IGZO 2:1:2	2.30±0.08
IGZO 2:1:1	2.26±0.06
IGZO 3:1:1	2.28±0.06

Thickness and E_{Opt} of thin films

Table S8. Combustion IGZO films' thickness measured by spectroscopic ellipsometry.

# Layers	Thickness (nm) In:Ga:Zn Ratio			
	**1:1:1**	**2:1:2**	**2:1:1**	**3:1:1**
**1**	13.6 ± 0.1	14.2 ± 0.1	13.7 ± 0.1	14.2 ± 0.1
**2**	28.1 ± 0.2	27.4 ± 0.3	24.0 ± 0.6	26.6 ± 0.2
**3**	38.8 ± 0.1	38.2 ± 0.3	36.2 ± 0.3	35.9 ± 0.4

Table S9. Bandgap energy (Eg) of combustion IGZO thin films determined by spectroscopic ellipsometry.

# Layers	Bandgap Energy (eV) In:Ga:Zn Ratio			
	**1:1:1**	**2:1:2**	**2:1:1**	**3:1:1**
**1**	3.71	3.39	3.41	3.52
**2**	3.70	3.42	3.39	3.40
**3**	3.66	3.46	3.42	3.41

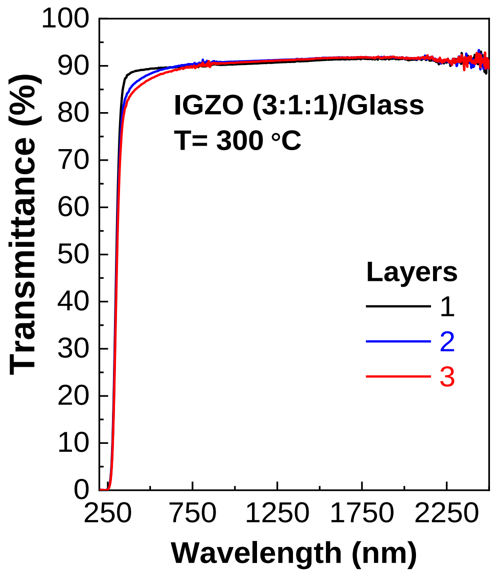

Figure S2. Transmittance measurements of combustion of 1-, 2- and 3-layer IGZO 3:1:1.

AFM characterisation

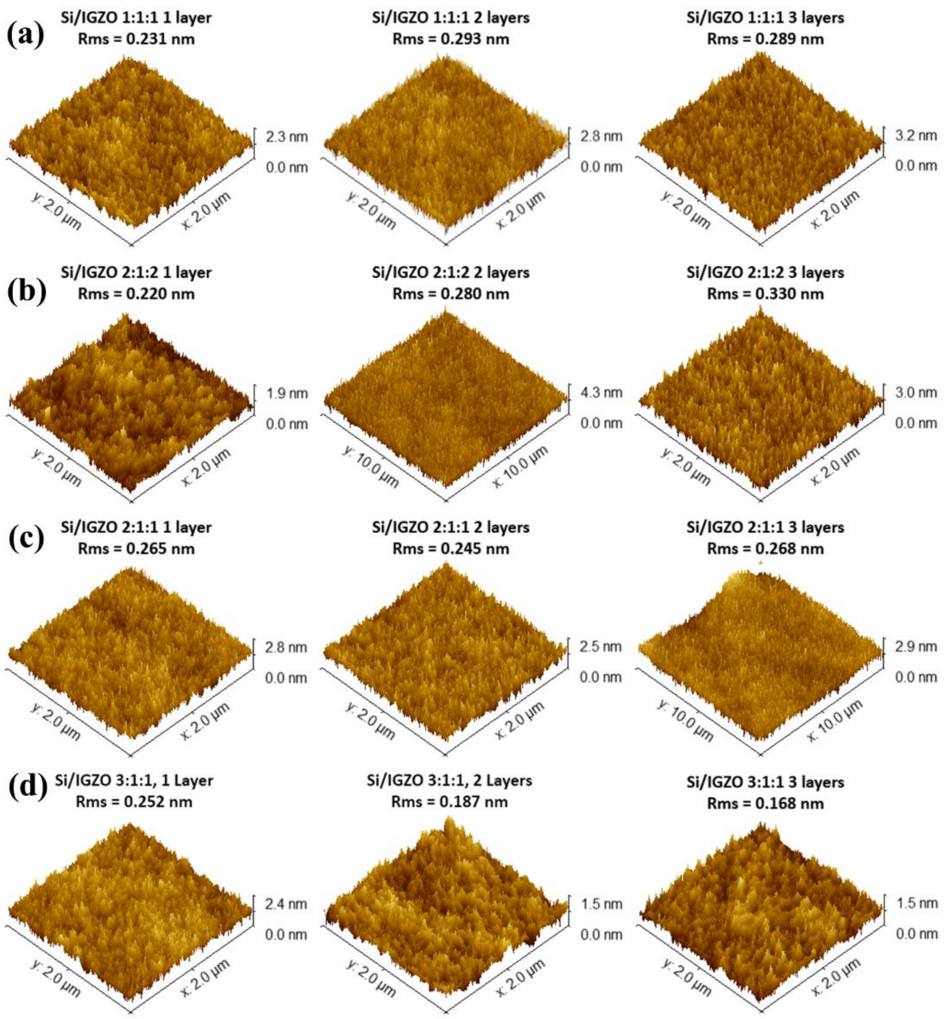

Figure S3. AFM deflection images (2×2 μm²) of 1-, 2- and 3-layer IGZO thin films with different In:Ga:Zn ratio (**a**) 1:1:1, (**b**) 2:1:2, (**c**) 2:1:1 and d) 3:1:1.

XPS depth-profile analysis

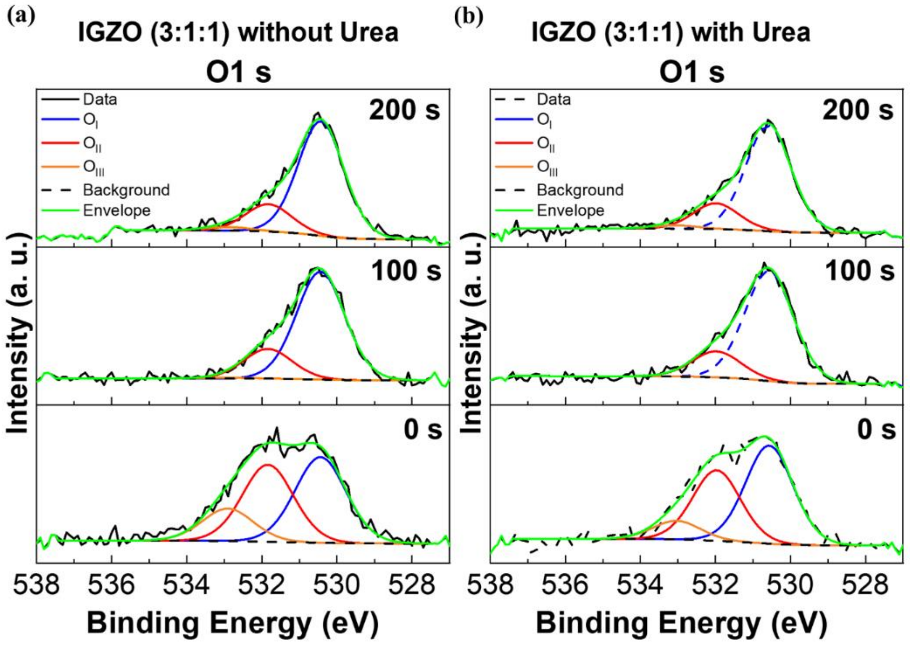

Figure S4. Deconvoluted O 1s spectra of the XPS depth profile after 0 s, 100 s and 200 s argon cluster etching.

Electrical characterisation of devices

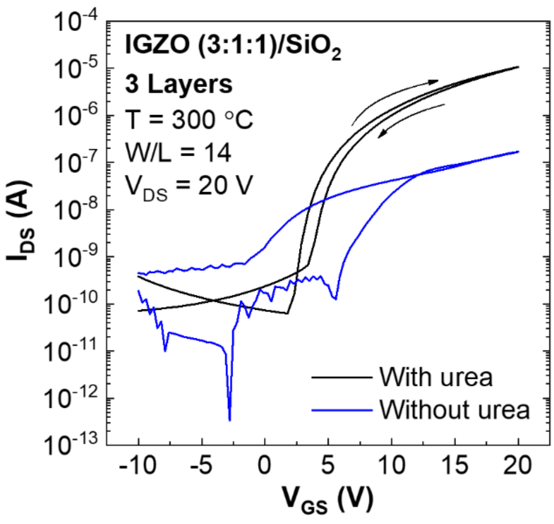

Figure S5. Transfer characteristics of 3-layer 3:1:1 IGZO TFTs produced using IGZO precursor solutions with and without urea as fuel.

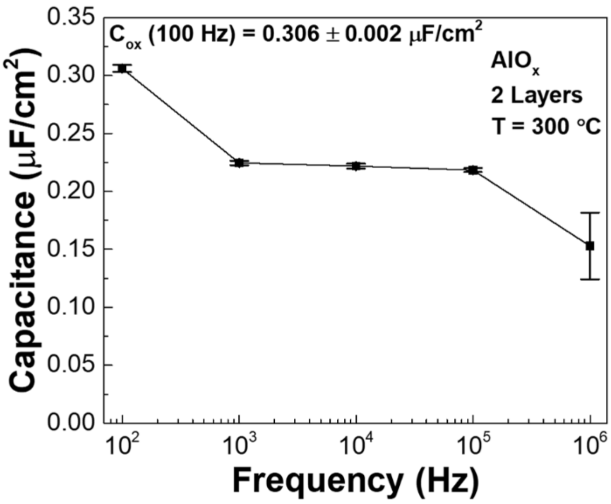

Figure S6. Capacitance-frequency measurements of Si/solution-based AlO_x/Al MIS device.

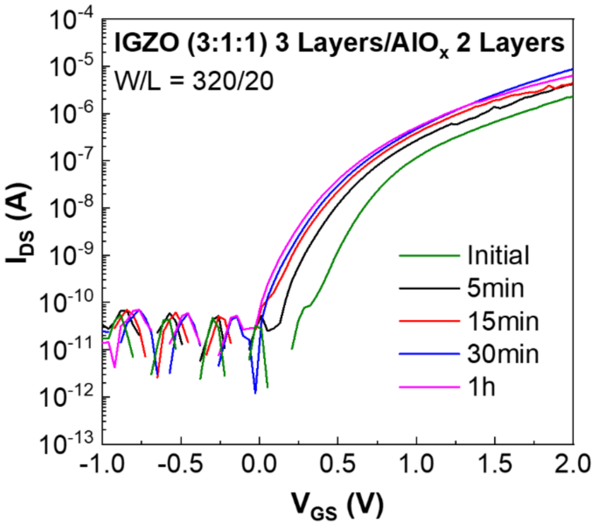

Figure S7. Transfer characteristics of 3-layer IGZO (3:1:1)/AlO_x TFT when a positive gate bias stress (PBS) of 0.5 $MV{\cdot}cm^{-1}$ is aplied over time.

References

1. Branquinho, R.; Santa, A.; Carlos, E.; Salgueiro, D.; Barquinha, P.; Martins, R.; Fortunato, E. Solution Combustion Synthesis: Applications in Oxide Electronics. In *Developments in Combustion Technology*; Konstantinos, K. G., Skvaril, J., Eds.; InTech, 2016; pp 397–417. https://doi.org/10.5772/64761.
2. Jain, S. R.; Adiga, K. C.; Pai Verneker, V. R. A New Approach to Thermochemical Calculations of Condensed Fuel-Oxidizer Mixtures. *Combust. Flame* **1981**, *40* (C), 71–79. https://doi.org/10.1016/0010-2180(81)90111-5.
3. Xie, M.; Wu, S.; Chen, Z.; Khan, Q.; Wu, X.; Shao, S.; Cui, Z. RSC Advances Performance Improvement for Printed Indium Gallium Zinc Oxide Thin- Fi Lm Transistors with a Preheating Process †. *RSC Adv.* **2016**, *6*, 41439–41446. https://doi.org/10.1039/C6RA01776B.
4. Nomura, K.; Kamiya, T.; Hosono, H. Effects of Diffusion of Hydrogen and Oxygen on Electrical Properties of Amorphous Oxide Semiconductor, In-Ga-Zn-O. *ECS J. Solid State Sci. Technol.* **2012**, *2* (1), P5–P8. https://doi.org/10.1149/2.011301jss.
